# Inflammatory Biomarkers in Coronary Artery Ectasia: A Systematic Review and Meta-Analysis

**DOI:** 10.3390/diagnostics12051026

**Published:** 2022-04-19

**Authors:** Dimitrios A. Vrachatis, Konstantinos A. Papathanasiou, Dimitrios Kazantzis, Jorge Sanz-Sánchez, Sotiria G. Giotaki, Konstantinos Raisakis, Andreas Kaoukis, Charalampos Kossyvakis, Gerasimos Deftereos, Bernhard Reimers, Dimitrios Avramides, Gerasimos Siasos, Michael Cleman, George Giannopoulos, Alexandra Lansky, Spyridon Deftereos

**Affiliations:** 1 Medical School, National and Kapodistrian University of Athens, 11527 Athens, Greece; dvrachatis@gmail.com (D.A.V.); kpapathanasiou91@gmail.com (K.A.P.); dkaza91@yahoo.gr (D.K.); sotiria.giotaki@yahoo.com (S.G.G.); ger_sias@hotmail.com (G.S.); 2 Division of Cardiology, Hospital Universitario y Politécnico La Fe, 46026 Valencia, Spain; sjorge4@gmx.com; 3 Centro de Investigacion Biomédica en Red (CIBERCV), 28029 Madrid, Spain; 4 Deparment of Cardiology, General Hospital of Athens “G.Gennimatas”, 11527 Athens, Greece; kraisakis@yahoo.co.uk (K.R.); andreaskaoukis@yahoo.gr (A.K.); ckossyvakis@gmail.com (C.K.); gerasimosd@gmail.com (G.D.); d_avramides@yahoo.com (D.A.); 5 Humanitas Clinical and Research Center IRCCS, 20089 Milan, Italy; bernhard.reimers@humanitas.it; 6 Section of Cardiovascular Medicine, Department of Internal Medicine, Yale School of Medicine, New Haven, CT 06510, USA; michael.cleman@yale.edu (M.C.); alexandra.lansky@yale.edu (A.L.); 7 Medical School, Aristotle University of Thessaloniki, 54124 Thessaloniki, Greece; ggiann@med.uoa.gr

**Keywords:** coronary artery ectasia, inflammation, biomarkers, neutrophil lymphocyte ratio

## Abstract

Isolated coronary artery ectasia (CAE) is a relatively rare clinical entity, the pathogenesis of which is poorly understood. More and more evidence is accumulating to suggest a critical inflammatory component. We aimed to elucidate any association between neutrophil to lymphocyte ratio and coronary artery ectasia. A systematic MEDLINE database, ClinicalTrials.gov, medRxiv, Scopus and Cochrane Library search was conducted: 50 studies were deemed relevant, reporting on difference in NLR levels between CAE patients and controls (primary endpoint) and/or on high-sensitive CRP, IL-6, TNF-a and RDW levels (secondary endpoint), and were included in our final analysis. (PROSPERO registration number: CRD42021224195). All inflammatory biomarkers under investigation were found higher in coronary artery ectasia patients as compared to healthy controls (NLR; SMD = 0.73; 95% CI: 0.27–1.20, hs-CRP; SMD = 0.96; 95% CI: 0.64–1.28, IL-6; SMD = 2.68; 95% CI: 0.95–4.41, TNF-a; SMD = 0.50; 95% CI: 0.24–0.75, RDW; SMD = 0.56; 95% CI: 0.26–0.87). The main limitations inherent in this analysis are small case-control studies of moderate quality and high statistical heterogeneity. Our findings underscore that inflammatory dysregulation is implicated in coronary artery ectasia and merits further investigation.

## 1. Introduction

Coronary artery ectasia (CAE) is defined as dilation of the coronary arteries exceeding one third of vessel length with an abnormal diameter of the ectatic segment counting more than 1.5 times the diameter of a normal adjacent segment [1]. The incidence of CAE is reported in 1% to 5% of patients undertaking coronary angiography for coronary artery disease (CAD) evaluation [1]. Regarding etiology, rheumatologic disorders, systemic inflammatory diseases, congenital and iatrogenic origin have all been implicated, while atherosclerosis is reported as accounting for more than 50% of cases [1]. Isolated CAE (iCEA) is the term employed to describe cases with no apparent etiologic factor and accounts for up to 10% of total cases [2]. 

The exact pathophysiology of iCAE is poorly understood: inflammation, platelet activation, endothelial dysfunction, microvascular dysfunction, slow flow and vascular remodeling have all been suggested to play a role [1,2,3]. Available evidence implies that iCAE is not a mere variant of CAD; indeed diabetes is negatively associated with CAE and studies pinpoint a critical inflammatory component [4,5]. The right coronary artery is the most frequently affected vessel, while angina pectoris represents the major clinical complaint [1,6]. Long term outcomes of CAE are not well defined [3]. 

Recently, data from two long-term follow up studies demonstrated that (i) patients with iCAE in Northern Europe have increased cardiovascular mortality as compared to healthy controls [7] and (ii) angiographic extent of CAE and sluggish coronary flow are independent predictors of future acute coronary events in a large North American cohort [8]. Interestingly, both studies suggested that their findings were independent of cardiovascular risk factors and CAD history. 

Turbulent slow flow within dilated coronaries may lead to platelet activation, thrombosis and eventually acute coronary syndrome [2]. The aforementioned data in parallel with no established treatment guidelines pose an urgent need for further research concerning pathogenesis [2,3]. Neutrophil to lymphocyte ratio (NLR) is an inexpensive and readily accessible biomarker with established utility in cardiology [9,10,11,12,13] as well as infections [14,15], rheumatologic syndromes [16,17,18,19], solid tumors [20] and renal failure [21,22].

Here, we aimed to conduct a meta-analysis investigating the potential association between NLR and iCAE. Additionally, we evaluated data concerning other inflammatory biomarkers such as TNF-α, IL-6, high-sensitive CRP and red cell distribution width (RDW). 

## 2. Methods

### 2.1. Data Sources and Search Strategy

A meta-analysis of observational studies was performed according to the Preferred Reporting Items for Systematic Reviews and Meta-Analyses 2009 guidelines [23]. Two reviewers (KP, DK) independently identified the relevant studies by an electronic search of the MEDLINE database, Scopus, ClinicalTrials.gov, medRxiv and Cochrane Library from inception to 24 March 2022. The following search query was used: ((coronary ectasia) OR (coronary artery ectasia) OR (ectasia) OR (ectatic)) AND ((neutrophil lymphocyte ratio) OR (neutrophil) OR (lymphocyte) OR (NLR) OR (markers) OR (biomarker) OR (prediction) OR (predictive)) (Supplementary Table S1; Search Strings) Articles cited in the reference lists of initially identified articles by this query were reviewed in order to identify any supplemental studies (“snowball procedure”). The final list of eligible articles was filtered manually to exclude duplicates. The protocol for this study was registered in PROSPERO (registration number: CRD42021224195).

### 2.2. Inclusion and Exclusion Criteria

In order for a study to be eligible it had to fulfill the following criteria: (1) evaluated a CAE population and a healthy control population or patients with coronary artery disease for comparison; (2) employed a clearly stated definition of CAE; (3) evaluated the level of serum inflammatory biomarkers in patients and controls. Studies were excluded if they were: (1) not published in English language; (2) not reporting mean or median values and standard deviation of NLR; (3) case reports; (4) evaluating coronary artery ectasia related to atherosclerosis (when no distinction was made between isolated CAE cases and atherosclerosis related cases) or other secondary condition; (5) not employing a clear definition regarding CAE diagnosis.

### 2.3. Data Extraction

Data were independently extracted and reviewed from each study by two reviewers (KP, DK). Any discrepancy between data extractions was resolved by discussion or a third reviewer (DV). The following data were extracted: first author, year of publication, country, study design (prospective/retrospective), number of patients and controls, patient demographics, matching criteria and descriptive statistics of inflammatory biomarkers in patients and controls.

### 2.4. Quality Assessment

Quality of the included studies was conducted via the Newcastle-Ottawa Scale (NOS) [24], in which a study was judged on three categories: selection, comparability, and exposure/outcome. A nine-point scale of the NOS (range, 0–9 points) was eventually used for the evaluation. Two authors (KP, DK) discussed the implementation of this quality assessment tool and independently assessed the studies. Studies were defined as high quality if they had more than seven points, as medium quality if they had between four and six points, and as poor quality if they had fewer than four points.

### 2.5. Outcomes of Interest

The pre-specified primary endpoint was difference in NLR levels between CAE patients and controls. Secondary endpoints were high-sensitive CRP, IL-6, TNF-a and RDW. Each endpoint was assessed and measured according to the definitions reported in the original study protocols (Supplementary Table S2).

### 2.6. Statistical Analysis

The descriptive statistics were described as mean ± SD. For continuous outcomes the standardized mean difference (SMD) with 95% CI was used as the summary statistic and trial-specific data were pooled with the inverse-variance random-effects method. When mean and standard deviation were not available, they were derived from sample size, median and range based on a method previously described by Wan et al. [25]. The presence of heterogeneity among studies was evaluated with the Cochran Q chi-square test with *p* ≤ 0.1 considered to be of statistical significance, estimating the between-studies variance tau-square, and using the I^2^ test to evaluate inconsistency. I^2^ values of 25%, 50% and 75% were assigned adjectives of low, moderate and high heterogeneity. A leave-one-out sensitivity analysis was performed by iteratively removing one study at a time to confirm that our results were not driven by any single. In addition, a sensitivity analysis by calculating SMD using the inverse-variance fixed-effects method was performed for all outcomes of interest. Publication biases were assessed with Egger test and by visual inspection with funnel plots. All analyses were performed with Review Manager, version 5.3 (Copenhagen: The Nordic Cochrane Centre, The Cochrane Collaboration, 2014) and Stata, version 13 (StataCorp LP, College Station, TX, USA). The guidelines summarized in the MOOSE statements were followed [26]. 

## 3. Results

**Figure 1:**
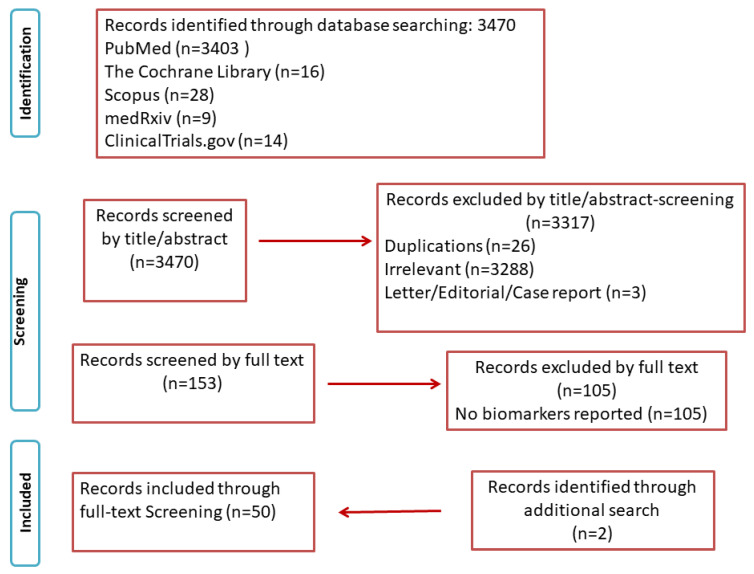
PRISMA flowchart.

The electronic database search identified 3470 studies. After screening of all titles and abstracts of potentially relevant articles, a total of 50 studies met the inclusion criteria (Figure 1). 

The study characteristics of the included studies are presented in Supplementary Table S3. 

### 3.1. Clinical Results

#### 3.1.1. Neutrophil to Lymphocyte Ratio

**Figure 2:**
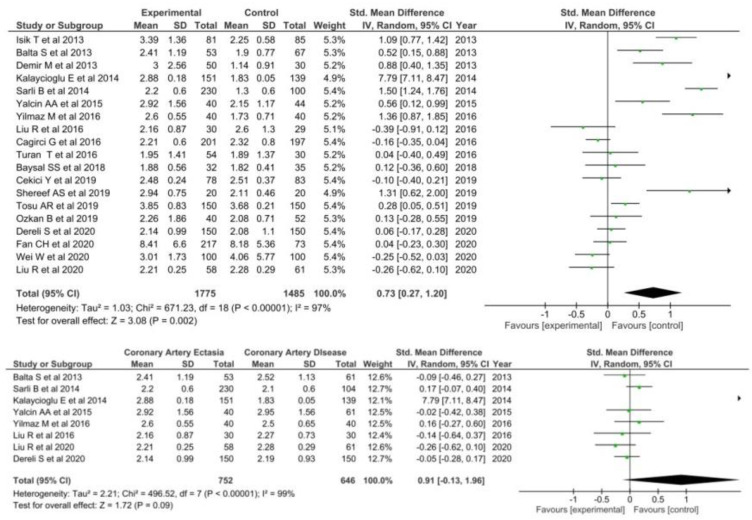
NLR in CAE patients versus controls (**top**) and CAE versus CAD patients (**bottom**).

A total of nineteen studies [27,28,29,30,31,32,33,34,35,36,37,38,39,40,41,42,43,44,45] involving 1775 patients with CAE and 1485 healthy controls were included comparing NLR levels in CAE with healthy controls. NLR was significantly higher in patients with CAE (SMD = 0.73; 95% CI: 0.27–1.20, I^2^ = 97%) as compared to healthy controls (Figure 2). 

Regarding the comparison of NLR levels between patients with CAE and CAD, eight studies were deemed eligible [27,28,35,37,38,39,41,42]. NLR levels were not significantly higher in CAE patients (SMD = 0.91; 95% CI: −0.13–1.96, I^2^ = 99%). 

#### 3.1.2. High Sensitivity CRP

**Figure 3:**
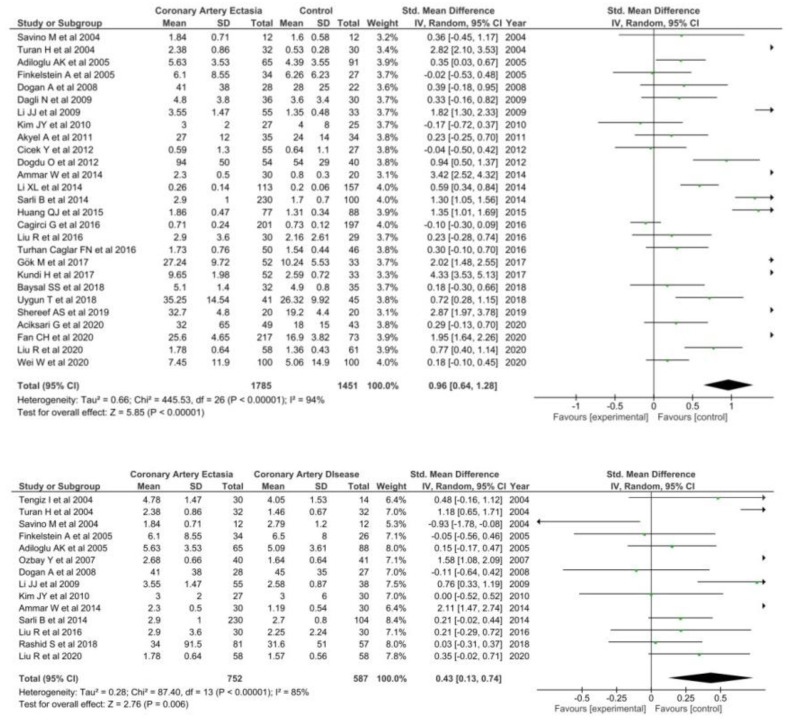
hs-CRP in CAE patients versus controls (**top**) and CAE versus CAD patients (**bottom**).

Twenty seven studies [30,33,34,35,36,37,38,45,46,47,48,49,50,51,52,53,54,55,56,57,58,59,60,61,62,63,64,65] involving 1785 patients with CAE and 1451 healthy controls were included in the meta-analysis. Hs-CRP levels were significantly higher in patients with CAE (SMD = 0.96; 95% CI: 0.64–1.28, I^2^ = 94%) as compared with healthy controls (Figure 3). 

High-sensitive CRP levels were also significantly higher in patients with CAE as compared with patients with CAD (SMD = 0.43; 95% CI: 0.13–0.74, I^2^ = 85%). 

#### 3.1.3. IL-6

**Figure 4:**
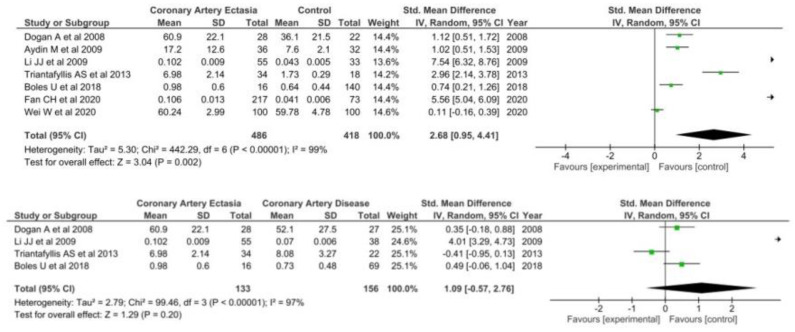
IL-6 in CAE patients versus control (**top**) and CAE versus CAD patients (**bottom**).

Seven articles [29,44,51,64,65,66,67] were included when comparing IL-6 levels between CAE patients and healthy controls including a total of 904 patients. IL-6 levels were significantly higher in patients with CAE (SMD = 2.68; 95% CI: 0.95–4.41, I^2^ = 99%) as compared to healthy controls (Figure 4). 

Conversely, IL-6 levels were not found to be higher in CAE patients than CAD patients (SMD = 1.09; 95% CI: −0.57–2.76, I^2^ = 97%).

#### 3.1.4. TNF-a

**Figure 5:**
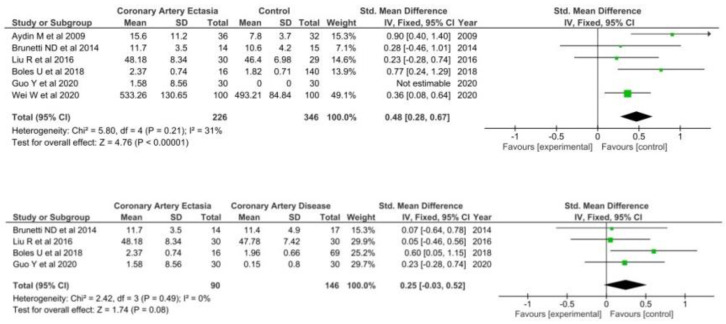
TNF-a in CAE patients versus controls (**top**) and CAE versus CAD patients (**bottom**).

A total of six studies [35,44,65,67,68,69] including 226 patients with CAE and 346 healthy controls were included when comparing TNF-a levels in the two groups. TNF-a levels were significantly higher in patients with CAE (SMD = 0.50 95% CI: 0.24–0.75, I^2^ = 31) (Figure 5). 

However, in the four studies [35,67,68,69] comparing TNF-a levels between patients with CAE and CAD no differences were found (SMD = 0.25; 95% CI: −0.03–0.52, I^2^ = 0%). 

#### 3.1.5. Red Cell Distribution

**Figure 6:**
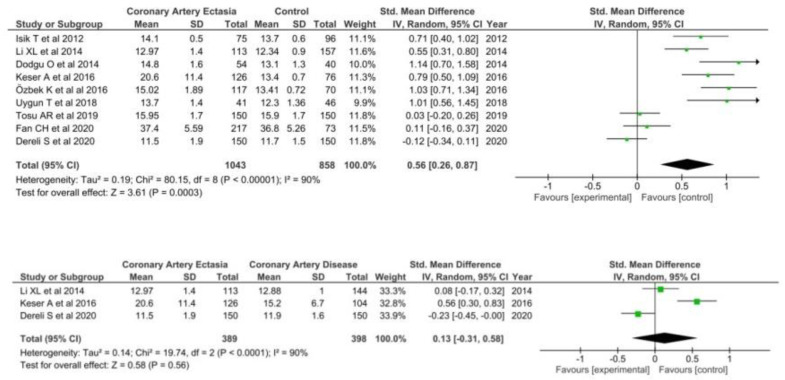
RDW in CAE patients versus controls (**top**) and CAE versus CAD patients (**bottom**).

A total of nine studies [29,38,43,50,61,63,70,71,72] comparing RDW in 1043 patients with CAE and 858 healthy controls were included in the meta-analysis. RDW was significantly higher in patients with CAE (SMD = 0.56; 95% CI: 0.26–0.87) as compared with healthy controls (Figure 6). 

A total of three studies [38,63,70] reported RDW levels on patients with CAE and CAD, including 787 patients in both groups. No differences were found in patients with CAE as compared with patients with CAD (SMD = 0.13; 95% CI: −0.31–0.58). 

### 3.2. Sensitivity Analyses

Compared to the main analysis, results remained unchanged after pooling the data using a fixed-effects model, as well as a leave-one-out sensitivity analysis (Supplementary Tables S5–S16). 

### 3.3. Risk of Bias Assessment

The quality assessment scores of the NOS are shown in Supplementary Table S4. Fourteen trials were of high quality and the remaining thirty-six were of moderate quality. 

#### Assessment of Publication Bias

Funnel-plot distributions of the pre-specified outcomes as well as Egger tests indicated absence of publication bias and small study effect for all the outcomes (Supplementary Figures S1–S10).

## 4. Discussion

The present study elucidates the pathophysiology of CAE and specifically suggests that inflammation is at least among the contributory factors. In particular, all investigated biomarkers (NLR, hs-CRP, IL-6, TNF-a and RDW) were shown to be elevated in CAE patients as compared to controls. Interestingly hs-CRP was also found to be higher in iCAE patients relative to CAD. Taking into account that all studies included in the analysis excluded patients suffering from inflammatory and infective disease, the above-mentioned findings imply that inflammation is an integral mechanistic link in iCAE pathogenesis and that, in a second level, inflammation may be more pronounced in patients suffering from CAE than CAD. IL-6, RWD and NLR levels were shown to be comparable between iCAE and CAD patients, thus hindering any definite conclusion regarding pathophysiologic differences. 

Pharmacologic management of CAE is still disputable, yet cardiovascular risk factor management and administration of aspirin and statins seem reasonable on the presence of atherosclerosis. The role of anticoagulants and angiotensin converting enzyme inhibitors mandate further evaluation [72]. Furthermore, immunomodulating agents may merit further investigation. Atherosclerosis is known to be driven by inflammatory perturbations [73,74]. Lately, anti-inflammatory treatments in cardiovascular disease have been focused on, showing benefit in large RCTs [75,76]. Besides, not only iCAE but also atherosclerosis-related CAE might be positively affected by anti-inflammatory medications. In which case a one-size-fits-all medication could potentially prove valuable in treating the vast majority of CAE patients (CAD-related and isolated cases) at risk of ACS development. 

Although not adequately tested in clinical studies, non-pharmacological approaches such as shock wave therapy and low level laser therapy might be beneficial in iCAE patients, since a growing body of pre-clinical data have already suggested their anti-inflammatory properties [77,78].

Percutaneous coronary intervention of iCAE patients suffering an acute coronary event is beyond the scope of this review, yet a limited number of published studies suggest higher mortality, target vessel revascularization and stent thrombosis as compared to patients with non-ectatic culprit vessels [79].

Further, the prognostic role of inflammatory biomarkers in iCAE needs further evaluation. Indeed, very few data regarding the association between NLR and CAE severity [80] are available. Yalcin AA. et al. [41] reported a positive correlation between CAE severity and NLR values, while Liu R. et al. have recently found that neither NLR nor hs-CRP are associated with Markis type [37]. 

In contrast, Sarli B. et al. found that both hs-CRP and NLR are independent predictors of disease severity [28]. Shereef AS. et al. suggested that hs-CRP and NLR are both related to Markis type in CAE patients—hs-CRP cut-off value above 2.35 mg/dl demonstrated 95% sensitivity in detecting CAE and NLR value above 2.65 has 95% specificity [36]. The same group of researchers reported that only hs-CRP is an independent predictor of CAE presence [36]. Kalaycioglu E. et al. proved that NLR is an independent predictor of both CAE presence and CAE type (severity) [39]. 

The role of NLR in CAE prediction has been addressed by a series of investigators. In particular, Cagirci G. et al. suggested that none of the examined biomarkers (TNF-α, IL-6, hs-CRP and NLR) may serve as an independent predictor of CAE presence [33]. Further, Cekici Y. et al. reached the same conclusion after finding a non-significant odds ratio for NLR [31]. Conversely, Demir M. et al. [45] and Yilmaz M. et al. proposed that NLR is an independent predictor of CAE [42]. Recently, Fan CH. et al. [29] published their finding suggesting that only IL-6 and hs-CRP can independently foretell CAE, while Tosu AR et al. reported that NLR is not an independent predictor in their study sample [43]. Additionally, Isik T. et al. found that, while NLR has only a modest sensitivity and specificity in identifying CAE patients (77% and 63%, respectively), it is still an independent predictor of CAE presence (odds ratio reported 6.03; *p* < 0.001) [40]. Consistently, Kalaycioglu E. et al. demonstrated that not only does NLR serve as an independent predictor of CAE presence but also it can discriminate CAE from obstructive CAD, as well as normal coronaries, when a cut-off value around 2 is applied [39]. 

A small but meticulous genome-wide association study indicated significant alterations in methylation levels of genes implicated in inflammation between CAE patients and controls [81] Additionally, emerging evidence suggests that diffuse CAE is associated with worse long-term outcomes [82] and NLR can predict acute coronary events in these susceptible patients [83]. Taken all together, the examination of NLR and other indices’ roles in identifying isolated CAE and its long-term outcomes seems reasonable and promising.

The present study has some limitations. First, this is a study-level meta-analysis providing average treatment effects. The lack of patient-level data prevented us from assessing the impact of baseline clinical characteristics on treatment effects. Second, results of this study were grounded on small case-control studies of moderate quality. In particular, comparability between groups may be inadequate, since adjustment for confounding factors was not part of the initial design in the majority of the studies. Third, data were derived from studies conducted mostly in one geographic location (Turkey), thus rendering any extrapolation to other populations challenging. Finally, we observed high heterogeneity in our findings, which can imply methodology issues such as different assays utilized for laboratory investigations, diverse population characteristics, timing of inflammatory indices measurement and adequacy of exclusion criteria evaluation. 

## 5. Conclusions

Patients with CAE as compared to normal coronary controls feature higher levels of inflammatory biomarkers. The role of these biomarkers in pathophysiology, CAE management and risk stratification merit further investigation.

## Supplementary Materials

The following supporting information can be downloaded at: https://www.mdpi.com/article/10.3390/diagnostics12051026/s1, Table S1: Search Strings, Table S2: Laboratory methods, Table S3: Studies characteristics. NOS; Newcastle-Ottawa scale, NA; not available, N; population. Table S4: Study quality according to Newcastle-Ottawa scale (NOS). Studies were defined as high quality if they had more than seven points, as medium quality if they had between four and six points, and as poor quality if they had fewer than four points, Table S5: Sensitivity Analysis; NLR in Coronary Artery Ectasia versus controls, Table S6: Sensitivity Analysis; NLR in Coronary Artery Ectasia versus Coronary Artery Disease, Table S7: Sensitivity Analysis; CRP in Coronary Artery Ectasia versus controls, Table S8: Sensitivity Analysis; CRP in Coronary Artery Ectasia versus Coronary Artery Disease, Table S9: Sensitivity Analysis; IL-6 in Coronary Artery Ectasia versus controls, Table S10: Sensitivity Analysis; IL-6 in Coronary Artery Ectasia versus Coronary Artery Disease, Table S11: Sensitivity Analysis; RDW in Coronary Artery Ectasia versus controls, Table S12: Sensitivity Analysis; RDW in Coronary Artery Ectasia versus Coronary Artery Disease, Table S13: Sensitivity Analysis; TNF-a in Coronary Artery Ectasia versus controls, Table S14: Sensitivity Analysis; TNF-a in Coronary Artery Ectasia versus Coronary Artery Disease, Table S15: Fixed Effects model results in CAE versus controls, Table S16: Fixed Effects model results in CAE versus CAD, Figure S1: Funnel plot for NLR CAE versus Controls (Egger test; *p* = 0.344), Figure S2: Funnel plot for NLR CAE versus CAD, Figure S3: Funnel plot for hs-CRP CAE versus Controls (Egger test; *p* = 0.071), Figure S4: Funnel plot for hs-CRP CAE versus CAD (Egger test; *p* = 0.169), Figure S5: Funnel plot for IL-6 CAE versus Controls, Figure S6: Funnel plot for IL-6 CAE versus CAD, Figure S7: Funnel plot for TNF-a CAE versus Controls, Figure S8: Funnel plot for TNF-a CAE versus CAD, Figure S9: Funnel plot for RDW CAE versus Controls, Figure S10: Funnel plot for RDW CAE versus CAD.
