# Supplementary material for: Inflammatory Biomarkers in Coronary Artery Ectasia: A Systematic Review and Meta-Analysis
Source: Diagnostics (Basel). 2022 Apr 19;12(5):1026. doi: 10.3390/diagnostics12051026 (PMC9140118; doi:10.3390/diagnostics12051026)
Supplement: Supplementary file 1 [file diagnostics-12-01026-s001.zip › Supp table 1-4.pdf]

Supplementary Table S1: Search Strings

**Database**	**Keywords/Search String**	**Filters**
PubMed	((coronary ectasia) OR (coronary artery ectasia) OR (ectasia) OR (ectatic)) AND ((neutrophil lymphocyte ratio) OR (neutrophil) OR (lymphocyte) OR (NLR) OR (markers) OR (biomarker) OR (prediction) OR (predictive))	none
Scopus	TITLE-ABS-KEY (coronary AND artery AND ectasia AND neutrophil AND lymphocyte AND ratio)	None
Cochrane Library	#1 coronary artery ectasia:ti,ab,kw	None
medRxiv	coronary artery ectasia	None
ClinicalTrials.gov	completed studies | coronary artery ectasia	completed studies

Supplementary Table S2: Laboratory methods

Author(Year)	***Laboratory Methods***				
	NLR	***hs-CRP***	***TNF-a***	***IL-6***	***RDW***
Aciksari G et al 2020		NA			
Adiloglu et al 2005		particle-enhanced immunonephelometry Dade Behring; Schwalbach, Germany		ELISA (Bio-Source International; Camarillo, Calif, USA)	
Akyel A et al 2011		NA			
Ammar W et al 2014		Dade Behring Inc., Marburg, Germany			
Aydin M et al 2009			ELISA Biosource International Inc. (Camarillo, Calif, USA)	ELISA (Biosource International Inc. Camarillo,Calif, USA)	
Balta S et al 2013	automatic blood counter LH 780 analyzer (Beckman Coulter Inc, Miami, Florida).				
Baysal SS et al 2018	NA	NA			
Boles U et al 2018			ELISA electrochemiluminescent detection method (MesoScale Discovery, Gaithersburg, MD, USA)	ELISA ectrochemiluminescent detection method (MesoScale Discovery, Gaithersburg, MD, USA)	
Brunetti ND et al 2014			Immulite, Siemens, Deerfield, IL, USA		
Cagirci G et 2016	Coulter LH 780 Hematology Analyzer impedance and optic scatter method	spectrophotometrically enzymatic-colorimetric assay Architect C16000 Clinical Chemistry Analyzer (Abbott Inc., Abbott Park, IL, USA)	NA	NA	
Cekici Y et al 2019	automated hematology analyzer (XE-1200 Sysmex, Kobe, Japan)				
Cicek Y et al 2012		Architect C16200 integrated systems (Abbott Diagnostics)			

Dagli N et al 2009		high-sensitivity immunonephelometry assay (Dade Behring Marburg, Germany)			
Demir M et al 2013	CELL-DYN 3700 (Abbott) impedance and optic scatter method				
Dereli S et al 2020	automated blood cell counter (Beckman Coulter Analyzer)				automated blood cell counter (Beckman Coulter Analyzer)
Dogan A et al 2008		nephelometer (Dade Behring Inc., Marburg, Germany)		ELISA (Biosource Europe SA, Nivelles, Belgium)	
Dogdu O et al 2012		NA			NA
Fan CH et al 2020	Horiba ABX 80 Diagnostics (ABX pentra Montpellier,France)	enzymatic colorimetric method automatic biochemical analyzer (Roche Cobas c702)		enzymatic colorimetric methods automatic biochemical analyzer (Roche Cobas c702)	Horiba ABX 80 Diagnostics (ABX pentra Montpellier,France)
Finkelstein A et al 2005		nephelometer (Dade Behring Inc., Marburg, Germany)			
Gök M et al 2017		ELISA			
Guo Y et al 2020			ELISA (BOSTER, Wuhan, China)		
Huang QJ et al 2014		NA			
Isik T et al 2012					Coulter LH 780 Hematology Analyzer (Beckman Coulter Ireland Inc, Mervue, Galway, Ireland)
Isik T et al 2013	Coulter LH 780 Hematology Analyzer (Beckman Coulter Ireland Inc. Mervue, Galway, Ireland)				
Kalaycioglu E et al 2014	automated haematology analyser Advia 2120 (Siemens)				

Keser A et al 2016					automatic blood cell counting instrument (K-X-21N auto analyzer; Sysmex Corp, Kobe, Japan)
Kim JY et al 2010		NA			
Kundi H et al 2017		automatised analyser			
Li JJ et al 2009		immunoturbidometry (Beckmann Assay 360, Bera, CA, USA)		commercial assay kit (Quantikine human IL-6, R&D System)	
Li XL et al 2014		immunoturbidometry (Beckmann Assay 360, Bera,CA, USA)			automated hematology analyzer XE-1200 (Sysmex, Kobe, Japan)
Liu R et al 2016	NA	NA	ELISA (Neobioscience Technology Co.,Ltd, Beijing, China)		
Liu R et al 2020	NA	NA			
Ozbay Y et al 2007		Dade Behring BN II (Germany)			
Özbek K et al 2016					Sysmex K-X-21N auto-analyzer
Ozkan B et al 2019	NA				
Rahimi B et al2020	Coulter® LH 780 automated hematology analyser electrical impedance method (Beckman Coulter Inc., Brea, CA, USA)				
Rashid S et al 2018		Beckman Coulter AU48			
Sarli B et al 2014	Cell-Dyn 3700 System; Abbot, Abbott Park, Illinois	BN-II nephelometer (Siemens, Marburg, Germany)			
Savino M et al 2006		highsensitivity, latex-enhanced immunonephelometric assay (Dade Behring BN II analyzer, Dade Behring, Germany)			
Shereef AS et al 2019	NA	NA			
Tengiz I et al 2004		latex particle-enhanced immunoturbidimetric assay			

Tosu AR et al 2019	Sysmex XT-1800i Hematology Analyzer device (Sysmex Corporation, Kobe, Japan)				Sysmex XT-1800i Hematology Analyzer device (Sysmex Corporation, Kobe, Japan).
Triantafyllis AS et al 2013				ELISA Bender MedSystems GmbH, Austria, Europe	
Turan H et al 2004		fluorescence polarization immunoassay (Abbott Diagnostics, Abbott Park,Illinois)			
Turan T et al 2016	BC-5800 auto hematology analyzer (Mindray Medical Electronics Co. Shenzhen, China)				
Turhan Caglar FN et al 2016		NA			
Uygun T et al 2018		ultra-high sensitive latex-based immunoassay method (Cobas integra, Roche Diagnostics, Mannheim,Germany)			LH 780 analyser (Beckman Coulter Inc, Miami, Florida)
Wei W et al 2020	NA	automatic biochemical immunoprotein analyzer (Shanghai UPPER Bio-tech Pharma Co., Ltd.,Shanghai, China)	ELISA Abcam ab225576	COBAS E411 chemiluminometer (Roche Diagnostics, Basel, Switzerland)	
Yalcin AA et al 2015	Mindray device BC-5800 (Mindray Bio-Medical Electronics Co Ltd, Shenzhen, China) optical laser method				
Yilmaz M et al 2016	Coulter LH 780 automated haematology analyser electrical impedance method (Beckman Coulter Inc., Brea, CA, USA				

Supplementary Table S3: Studies characteristics. NOS; Newcastle-Ottawa scale, NA; not available, N; population.

Author(Year)	*NOS stars*	*Journal*	*Study Design*	*Country*	*Total N*	*N CAE*	*N CAD*	*N Controls*
Aciksari G et al 2020	6	Cardiovasc J Afr	case-control	Turkey	92	49	NA	43
Adiloglu et al 2005	6	Tex Heart Inst J	case-control	Turkey	244	65	88	91
Akyel A et al 2011	6	Can J Cardiol	case-control	Turkey	70	35	NA	35
Ammar W et al 2014	7	The Egyptian Heart Journal	case-control	Egypt	80	30	30	20
Aydin M et al 2009	7	Mediators Inflamm	case-control	Turkey	68	36	NA	32
Balta S et al 2013	6	Angiology	case-control	Turkey	181	53	61	67
Baysal SS et al 2018	6	Eur Rev Med Pharmacol Sci	case-control	Turkey	67	32	NA	35
Boles U et al 2018	6	Int J Mol Sci	case-control	Sweden	225	16	69	140
Brunetti ND et al 2014	6	Atherosclerosis	case-control	Italy	46	14	17	15
Cagirci G et 2016	6	Korean Circ J	case-control	Turkey	398	201	NA	197
Cekici Y et al 2019	6	Acta Cardiol Sin	case-control	Turkey	161	78	NA	83
Cicek Y et al 2012	6	J Thromb Thrombolysis	case-control	Turkey	82	55	NA	27
Dagli N et al 2009	6	Heart Vessels	case-control	Turkey	66	36	NA	30
Demir M et al 2013	5	Cardiol Res	case-control	Turkey	80	50	NA	30
Dereli S et al 2020	*6*	Angiology	case-control	Turkey	450	150	150	150
Dogan A et al 2008	7	Coron Artery Dis	case-control	Turkey	77	28	27	22
Dogdu O et al 2012	6	Clin Appl Thromb Hemost	case-control	Turkey	94	54	NA	40
Fan CH et al 2020	7	BMC Cardiovascular Disorders	case-control	China	290	217	NA	73
Finkelstein A et al 2005	6	Atherosclerosis	case-control	Israel	89	34	26	27
Gök M et al 2017	6	Cardiovasc Endocrinol Metab	case-control	Turkey	85	52	NA	33
Guo Y et al 2020	6	BMC Cardiovasc Disord	case-control	China	90	30	30	30
Huang QJ et al 2014	7	Scand J Clin Lab Invest	case-control	China	167	79	NA	88
Isik T et al 2012	6	Clin Appl Thromb Hemost	case-control	Turkey	171	75	NA	96
Isik T et al 2013	7	Arch Turk Soc Cardiol	case-control	Turkey	166	81	NA	85

Kalaycioglu E et al 2014	6	Kardiol Pol	case-control	Turkey	405	151	128	139
Keser A et al 2016	6	Eur Rev Med Pharmacol Sci	case-control	Turkey	306	126	104	76
Kim JY et al 2010	7	Int J Cardiol	case-control	Korea	82	27	30	25
Kundi H et al 2017	6	Kardiol Pol	case-control	Turkey	87	52	NA	35
Li JJ et al 2009	7	Cytokine	case-control	China	126	55	38	33
Li XL et al 2014	6	Journal of Translational Medicine	case-control	China	414	113	144	157
Liu R et al 2016	6	Anatol J Cardiol	case-control	China	89	30	30	29
Liu R et al 2020	6	Turk J Med Sci	case-control	China	177	58	58	61
Ozbay Y et al 2007	6	Mediators Inflamm	case-control	Turkey	81	40	41	NA
Özbek K et al 2016	7	Biomed Res	case-control	Turkey	187	117	NA	70
Ozkan B et al 2019	7	Anatol J Cardiol	case-control	Turkey	92	40	NA	52
*Rahimi B et al*2020	5	J Res Clin Med	case-control	Iran	577	117	230	230
Rashid S et al 2018	6	J Coll Physicians Surg Pak	case-control	Pakistan	138	81	57	
Sarli B et al 2014	6	Angiology	case-control	Turkey	434	230	104	100
Savino M et al 2006	7	Int J Cardiol	case-control	Italy	36	12	12	12
Shereef AS et al 2019	6	J Indian coll cardiol	case-control	Egypt	60	20	20	20
Tengiz I et al 2004	6	Curr Control Trials Cardiovasc Med	case-control	Turkey	44	30	14	NA
Tosu AR et al 2019	7	Turk J Clin Lab	case-control	Turkey	300	150	NA	150
Triantafyllis AS et al 2013	7	Cytokine	case-control	Greece	74	34	22	18
Turan H et al 2004	6	Am J Catdio	case-control	Turkey	94	32	32	30
Turan T et al 2016	6	Angiology	case-control	Turkey	84	54	NA	30
Turhan Caglar FN et al 2016	6	Angiology	case-control	Turkey	96	50	NA	46
Uygun T et al 2018	6	Cytokine	case-control	Turkey	86	41	NA	45
Wei W et al 2020	6	J Thorac Dis	case-control	China	300	100	NA	100
Yalcin AA et al 2015	6	Clinical and Applied Thrombosis/Hemostasis	case-control	Turkey	198	40	62	44
Yilmaz M et al 2016	7	Journal of International Medical Research	case-control	Turkey	160	40	40	40

Supplementary Table S4: Study quality according to Newcastle-Ottawa scale (NOS). Studies were defined as high quality if they had more than seven points, as medium quality if they had between four and six points, and as poor quality if they had fewer than four points.

Author(Year)	*Selection*	*Comparability*	*Exposure*	*Study Quality*
Aciksari G et al 2020	3	0	3	Moderate
Adiloglu et al 2005	3	0	3	Moderate
Akyel A et al 2011	3	0	3	Moderate
Ammar W et al 2014	3	1	3	High
Aydin M et al 2009	3	1	3	High
Balta S et al 2013	4	0	2	Moderate
Baysal SS et al 2018	3	0	3	Moderate
Boles U et al 2018	3	0	0	Moderate
Brunetti ND et al 2014	3	0	3	Moderate
Cagirci G et 2016	3	0	3	Moderate
Cekici Y et al 2019	3	0	3	Moderate
Cicek Y et al 2012	3	0	3	Moderate
Dagli N et al 2009	3	0	3	Moderate
Demir M et al 2013	1	1	3	Moderate
Dereli S et al 2020	*3*	*0*	*3*	*Moderate*
Dogan A et al 2008	3	1	3	High
Dogdu O et al 2012	3	0	3	Moderate
Fan CH et al 2020	3	1	3	High
Finkelstein A et al 2005	3	0	3	Moderate
Gök M et al 2017	3	0	3	Moderate
Guo Y et al 2020	3	0	3	Moderate
Huang QJ et al 2014	3	0	3	High
Isik T et al 2012	3	0	3	Moderate

Isik T et al 2013	3	1	3	High
Kalaycioglu E et al 2014	3	0	3	Moderate
Keser A et al 2016	3	0	3	Moderate
Kim JY et al 2010	3	1	3	High
Kundi H et al 2017	3	0	3	Moderate
Li JJ et al 2009	3	1	3	High
Li XL et al 2014	3	0	3	Moderate
Liu R et al 2016	3	0	3	Moderate
Liu R et al 2020	3	0	3	Moderate
Ozbay Y et al 2007	3	0	3	Moderate
Özbek K et al 2016	3	1	3	High
Ozkan B et al 2019	4	0	3	High
*Rahimi B et al*2020	2	0	3	Moderate
Rashid S et al 2018	3	0	3	Moderate
Sarli B et al 2014	3	0	3	Moderate
Savino M et al 2006	3	1	3	High
Shereef AS et al 2019	3	0	3	Moderate
Tengiz I et al 2004	3	0	3	Moderate
Tosu AR et al 2019	3	1	3	High
Triantafyllis AS et al 2013	3	1	3	High
Turan H et al 2004	3	0	3	Moderate
Turan T et al 2016	3	0	3	Moderate
Turhan Caglar FN et al 2016	3	0	3	Moderate
Uygun T et al 2018	3	0	3	Moderate
Wei W et al 2020	3	0	3	Moderate
Yalcin AA et al 2015	3	0	3	Moderate
Yilmaz M et al 2016	3	1	3	High
