# Supplementary material for: Inflammatory Biomarkers in Coronary Artery Ectasia: A Systematic Review and Meta-Analysis
Source: Diagnostics (Basel). 2022 Apr 19;12(5):1026. doi: 10.3390/diagnostics12051026 (PMC9140118; doi:10.3390/diagnostics12051026)
Supplement: Supplementary file 1 [file diagnostics-12-01026-s001.zip › Supp Tables 5-16.pdf]

Supplementary Table S5: Sensitivity Analysis; NLR in Coronary Artery Ectasia versus controls

**Study Removed**	**SMD**	**95% CI**	**p-value overall effect**	I^2
Balta S et al 2013	0.75	0.26-1.24	0.003	97%
Baysal S et al 2018	0.77	0.28-1.25	0.002	97%
Cagirci G et al 2016	0.79	0.28-1.29	0.002	97%
Cekici Y et al 2019	0.78	0.29-1.27	0.002	97%
Demir M et al 2013	0.73	0.24-1.21	0.003	97%
Dereli S et al 2020	0.78	0.27-1.28	0.002	97%
Fan CH et al 2020	0.78	0.28-1.27	0.003	97%
Isik T et al 2013	0.71	0.23-1.20	0.004	97%
Kalaycioglu E et al 2014	0.36	0.09-0.63	0.009	92%
Liu R et al 2016	0.80	0.31-1.28	0.001	97%
Liu R et al 2020	0.79	0.30-1.28	0.001	97%
Ozkan B et al 2019	0.77	0.28-1.26	0.002	97%
Sarli B et al 2014	0.69	0.22-1.16	0.004	97%
Shereef AS et al 2019	0.70	0.23-1.18	0.004	97%
Tosu AR et al 2019	0.76	0.25-1.27	0.003	97%
Turan T et al 2016	0.77	0.29-1.26	0.002	97%
Wei W et al 2020	0.79	0.30-1.28	0.002	97%
Yalcin AA et al 2015	0.74	0.26-1.23	0.003	97%
Yilmaz M et al 2016	0.70	0.22-1.18	0.004	97%

Supplementary Table S6: Sensitivity Analysis; NLR in Coronary Artery Ectasia versus Coronary Artery Disease

Study Removed	SMD	95% CI	P-value overall effect	I^2
Balta S et al 2013	1.06	-0.13 – 2.26	0.08	99%
Kalaycioglu E et al 2014	-0.01	-0.13-0.12	0.93	0%
Sarli B et al 2014	1.03	-0.29-2.35	0.12	99%
Yalcin AA et al 2015	1.05	-0.13-2.24	0.08	99%
Yilmaz M et al 2016	1.03	-0.15-2.20	0.09	99%
Liu R et al 2016	1.07	-0.09-2.22	0.07	99%
Liu R et al 2020	1.09	-0.11-2.28	0.07	99%
DereliS et al 2020	1.06	-0.25-2.38	0.11	99%

Supplementary Table S7: Sensitivity Analysis; CRP in Coronary Artery Ectasia versus controls

Study Removed	SMD	95% CI	P-value overall effect	I^2
Aciksari G et al 2020	0.99	0.66-1.32	<0.00001	94%
Adiloglu AK et al 2005	0.99	0.65-1.33	<0.00001	94%
Akyel A et al 2011	0.99	0.66-1.32	<0.00001	94%
Ammar W et al 2014	0.88	0.57-1.20	<0.00001	94%
Baysal SS et al 2018	0.99	0.66-1.32	<0.00001	94%
Cagirci G et al 2016	1.01	0.68-1.33	<0.00001	93%
Cicek Y et al 2012	1.00	0.67-1.33	<0.00001	94%
Dagli N et al 2009	0.99	0.66-1.32	<0.00001	94%
Dogan A et al 2008	0.98	0.65-1.32	<0.00001	94%
Dogdu O et al 2012	0.96	0.63-1.30	<0.00001	94%
Fan CH et al 2020	0.92	0.60-1.23	<0.00001	93%
Finkelstein A et al 2005	1.00	0.67-1.33	<0.00001	94%
Gök M et al 2017	0.92	0.60-1.24	<0.00001	94%
Huang QJ et al 2015	0.95	0.62-1.28	<0.00001	94%
Kim JY et al 2010	1.01	0.68-1.33	<0.00001	94%
Kundi H et al 2017	0.84	0.54-1.14	<0.00001	93%
Li JJ et al 2009	0.93	0.60-1.25	<0.00001	94%
Li XL et al 2014	0.98	0.64-1.33	<0.00001	94%
Liu R et al 2016	0.99	0.66-1.32	<0.00001	94%
Liu R et al 2020	0.97	0.64-1.31	<0.00001	94%
Sarli B et al 2014	0.95	0.62-1.28	<0.00001	94%
Savino M et al 2004	0.98	0.65-1.31	<0.00001	94%
Shereef AS et al 2019	0.90	0.58-1.22	<0.00001	94%
Turan H et al 2004	0.89	0.58-1.21	<0.00001	94%
Turhan Caglar FN et al 2016	0.99	0.66-1.32	<0.00001	94%
Uygun T et al 2018	0.97	0.64-1.31	<0.00001	94%
Wei W et al 2020	1.00	0.66-1.33	<0.00001	94%

Supplementary Table S8: Sensitivity Analysis; CRP in Coronary Artery Ectasia versus Coronary Artery Disease

Study Removed	SMD	95% CI	P-value overall effect	I^2
Adiloglu AK et al 2005	0.46	0.12-0.79	0.008	86%
Ammar W et al 2014	0.32	0.06-0.59	0.02	79%
Dogan A et al 2008	0.47	0.15-0.79	0.004	86%
Finkelstein A et al 2005	0.47	0.15-0.79	0.005	86%
Kim JY et al 2010	0.46	0.14-0.79	0.005	86%
Li JJ et al 2009	0.41	0.08-0.73	0.01	86%
Liu R et al 2016	0.45	0.12-0.78	0.007	86%
Liu R et al 2020	0.44	0.10-0.78	0.01	86%
Ozbay Y et al 2007	0.34	0.06-0.62	0.02	81%
Rashid S et al 2018	0.47	0.13-0.80	0.006	86%
Sarli B et al 2014	0.45	0.10-0.81	0.01	86%
Savino M et al 2004	0.51	0.20-0.81	0.001	85%
Tengiz I et al 2004	0.43	0.11-0.75	0.009	86%
Turan H et al 2004	0.38	0.07-0.69	0.02	85%

Supplementary Table S9: Sensitivity Analysis; IL-6 in Coronary Artery Ectasia versus controls

**Study Removed**	**SMD**	**95% CI**	**P-value overall effect**	**I^2**
Aydin M et al 2009	2.97	0.88-5.06	0.005	99%
Boles U et al 2018	3.02	0.95-5.08	0.004	99%
Dogan A et al 2008	2.95	0.93-4.97	0.004	99%
Fan CH et al 2020	2.14	0.85-3.43	0.001	97%
Li JJ et al 2009	1.91	0.24-3.58	0.02	99%
Triantafyllis AS et al 2013	2.64	0.72-4.55	0.007	99%
Wei W et al 2020	3.12	1.16-5.08	0.002	98%

Supplementary Table S10: Sensitivity Analysis; IL-6 in Coronary Artery Ectasia versus Coronary Artery Disease

Study Removed	SMD	95% CI	P-value overall effect	I^2
Boles U et al 2018	1.30	-1.09-3.70	0.29	98%
Dogan A et al 2008	1.35	-1.04-3.74	0.27	98%
Li JJ et al 2009	0.14	-0.40-0.69	0.61	67%
Triantafyllis AS et al 2013	1.60	-0.49-3.70	0.13	97%

Supplementary Table S11: Sensitivity Analysis; RDW in Coronary Artery Ectasia versus controls

**Study Removed**	**SMD**	**95% CI**	**P-value overall effect**	**I^2**
Dereli S et al 2020	0.65	0.35-0.95	<0.0001	87%
Dodgu O et al 2014	0.50	0.19-0.81	0.002	90%
Fan CH et al 2020	0.62	0.29-0.96	0.0003	91%
Isik T et al 2012	0.55	0.21-0.88	0.001	91%
Keser A et al 2016	0.53	0.20-0.86	0.002	90%
Li XL et al 2014	0.57	0.22-0.92	0.002	91%
Tosu AR et al 2019	0.63	0.31-0.96	0.0002	91%
Uygun T et al 2018	0.51	0.20-0.83	0.002	90%
Özbek K et al al 2016	0.50	0.19-0.81	0.001	89%

Supplementary Table S12: Sensitivity Analysis; RDW in Coronary Artery Ectasia versus Coronary Artery Disease

Study Removed	SMD	95% CI	P-value overall effect	I^2
Dereli S et al 2020	0.32	-0.16 – 0.79	0.19	86%
Keser A et al 2016	-0.08	-0.38 – 0.22	0.60	68%
Li XL et al 2014	0.16	-0.61 – 0.94	0.68	95%

Supplementary Table S13: Sensitivity Analysis; TNF-a in Coronary Artery Ectasia versus controls

Study Removed	SMD	95% CI	P-value overall effect	I^2
Aydin M et al 2009	0.40	0.19-0.61	0.0002	0%
Boles U et al 2018	0.44	0.16-0.72	0.002	32%
Brunetti ND et al 2014	0.53	0.24-0.83	0.0004	45%
Guo Y et al 2020	0.50	0.24-0.75	0.0001	31%
Liu R et al 2016	0.56	0.27-0.86	0.0002	37%
Wei W et al 2020	0.57	0.23-0.92	0.001	34%

Supplementary Table S14: Sensitivity Analysis; TNF-a in Coronary Artery Ectasia versus Coronary Artery Disease

Author	SMD	95% CI	P-value overall effect	I^2
Boles U et al 2018	0.13	-0.19 – 0.45	0.44	0%
Brunetti ND et al 2014	0.28	-0.03 – 0.59	0.08	6%
Guo Y et al 2020	0.25	-0.11 – 0.62	0.18	17%
Liu R et al 2016	0.33	0.00 – 0.66	0.05	0%

Supplementary Table S15: Fixed Effects model results in CAE versus controls

Biomarker	SMD	95% CI	P
NLR	0.36	0.29 to 0.44	<0.00001
CRP	0.69	0.61-0.77	<0.00001
IL-6	1.40	1.22-1.59	<0.00001
RDW	0.42	0.32-0.51	<0.00001
TNF-a	0.48	0.28-0.67	<0.00001

Supplementary Table S16: Fixed Effects model results in CAE versus CAD

Biomarker	SMD	95% CI	P
NLR	0.23	0.11-0.35	0.001
CRP	0.36	0.25-0.47	<0.00001
IL-6	0.76	0.47-1.04	<0.00001
RDW	0.10	-0.04 – 0.24	0.18
TNF-a	0.25	-0.03 – 0.52	0.08
