# Supplementary material for: Inflammatory Biomarkers in Coronary Artery Ectasia: A Systematic Review and Meta-Analysis
Source: Diagnostics (Basel). 2022 Apr 19;12(5):1026. doi: 10.3390/diagnostics12051026 (PMC9140118; doi:10.3390/diagnostics12051026)

Supplementary Figure S1: Funnel plot for NLR CAE versus Controls (Egger test; p=0.344)

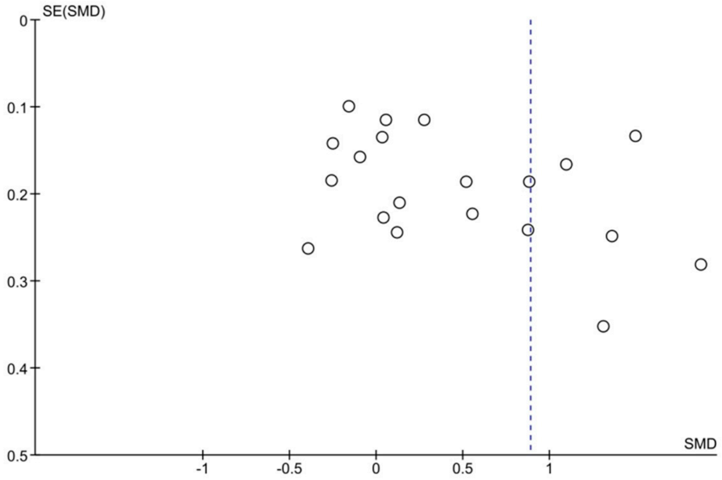

Supplementary Figure S2: Funnel plot for NLR CAE versus CAD

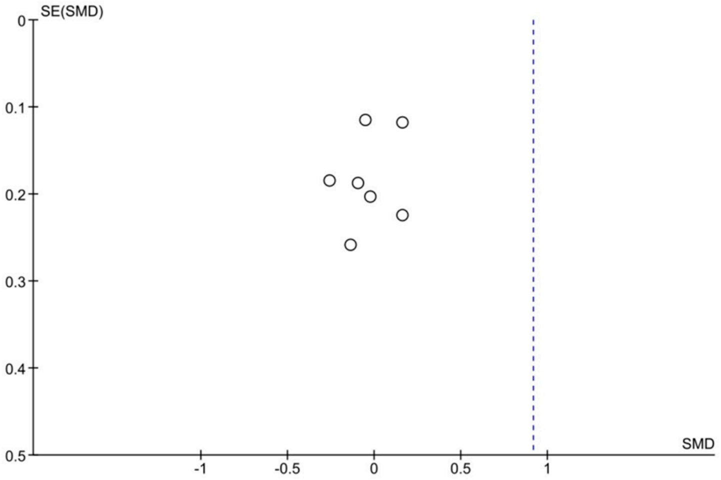

Supplementary Figure S3: Funnel plot for hs-CRP CAE versus Controls (Egger test; p=0.071)

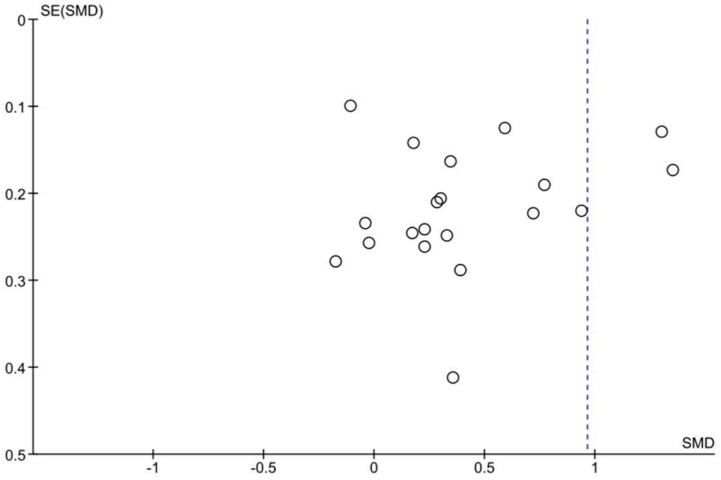

Supplementary Figure S4: Funnel plot for hs-CRP CAE versus CAD (Egger test; p=0.169)

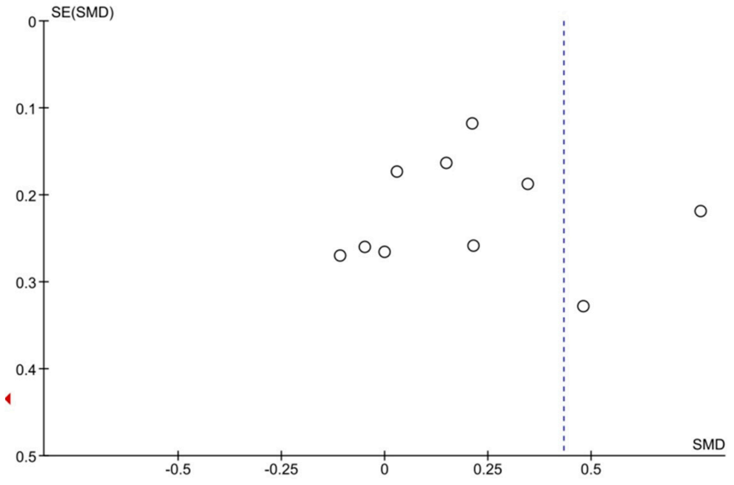

Supplementary Figure S5: Funnel plot for IL-6 CAE versus Controls

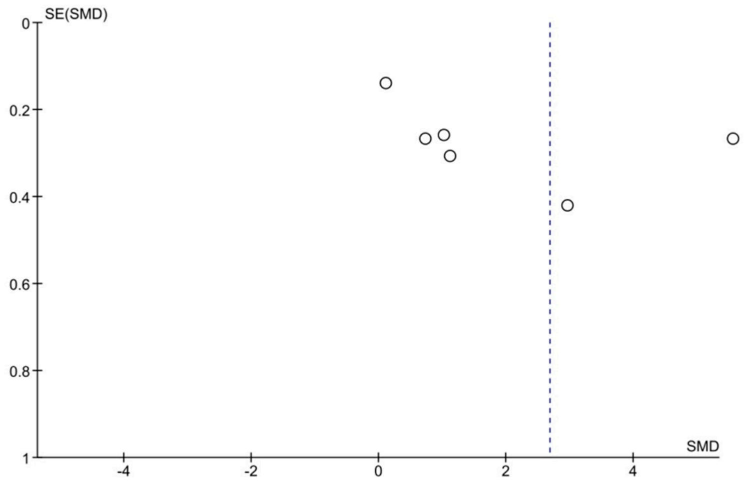

Supplementary Figure S6: Funnel plot for IL-6 CAE versus CAD

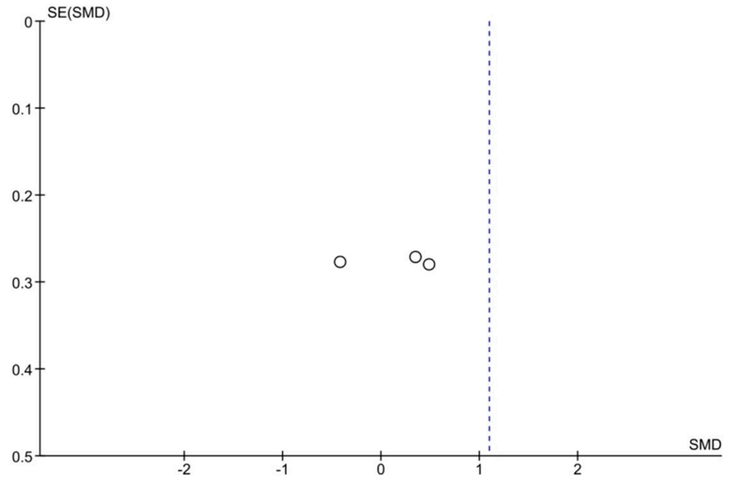

Supplementary Figure S7: Funnel plot for TNF-a CAE versus Controls

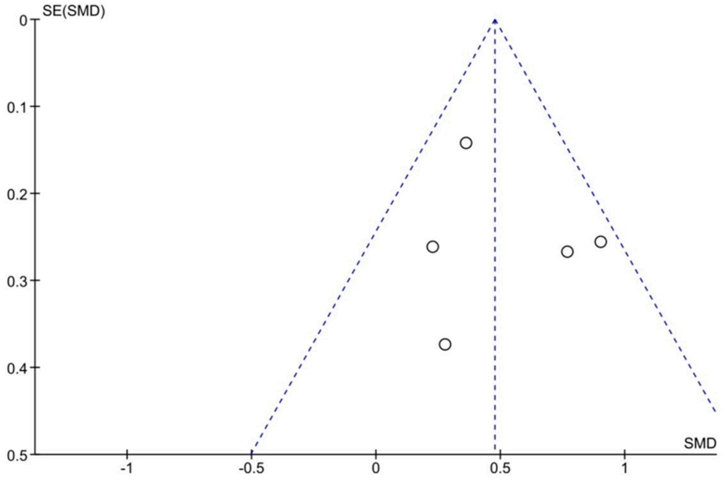

Supplementary Figure S8: Funnel plot for TNF-a CAE versus CAD

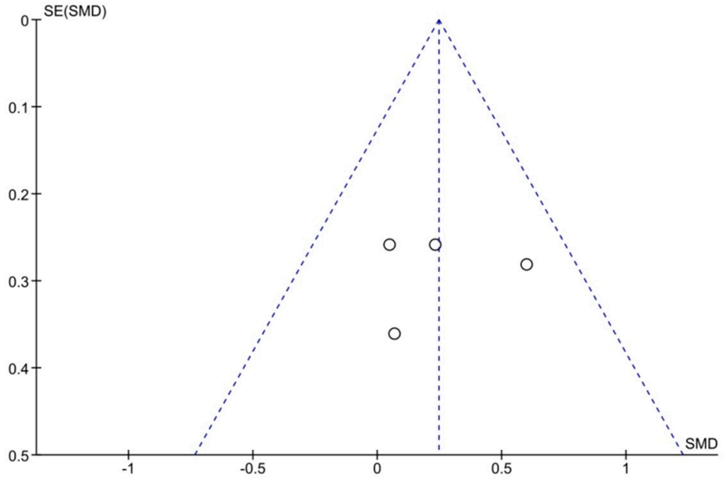

Supplementary Figure S9: Funnel plot for RDW CAE versus Controls

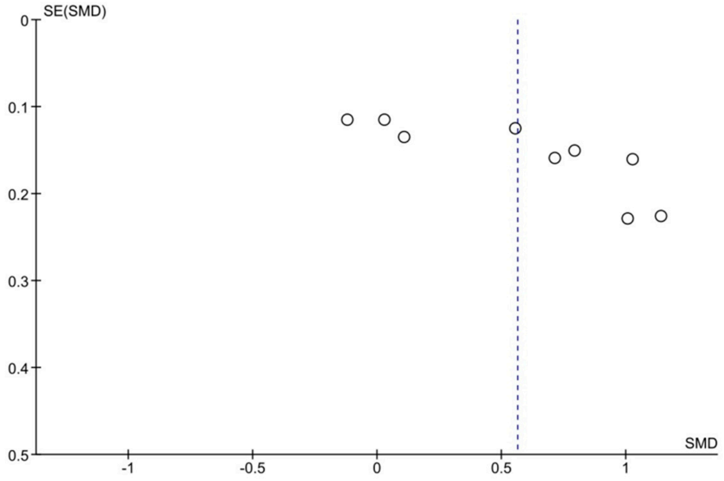

Supplementary Figure S10: Funnel plot for RDW CAE versus CAD

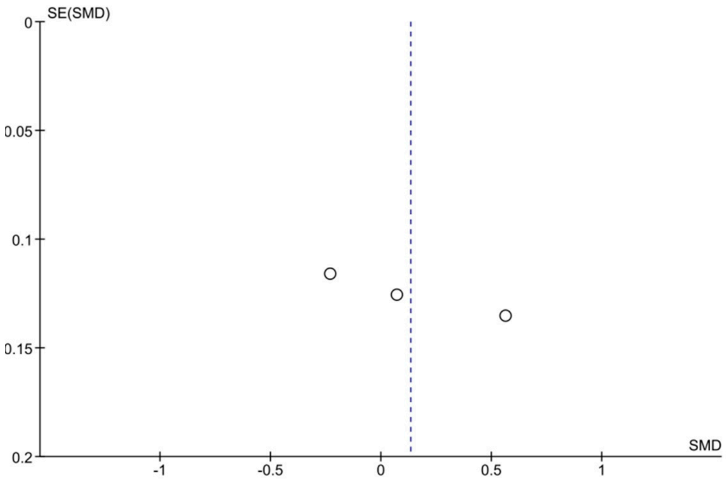

Supplement: Supplementary file 1 [file diagnostics-12-01026-s001.zip › Supplementary Figures.pdf]
